# Supplementary material for: Availability, scope and quality of monkeypox clinical management guidelines globally: a systematic review
Source: BMJ Glob Health. 2022 Aug 5;7(8):e009838. doi: 10.1136/bmjgh-2022-009838 (PMC9472169; doi:10.1136/bmjgh-2022-009838)
Supplement: Supplementary data [file bmjgh-2022-009838supp001.pdf]

Supplemental files

Table of Contents

S1: search strategy..2

Database search strategy..2-7

Scopus search strategy..7

Web of Science Core Collection search strategy..7

The WHO Global Index Medicus Regional Libraries search strategy..8

Database search results..8

Google Scholar search strategy..8-9

Grey Literature search strategy..9-11

S2: Data extraction..12

Data extraction form..12-15

S3: Figures and Tables..14

Figure S3.1 14

S1: SEARCH STRATEGY

S1.2: Database search strategy

Updated on 14/10/2021 by an Outreach Librarian at the Bodleian Health Care Libraries, University of Oxford. This review is part of a wider project evaluating the availability, quality, and inclusivity of clinical management guidelines for the management of high consequence infectious diseases (PROSPERO CRD42020167361).

Database: Medline (Ovid MEDLINE® Epub Ahead of Print, In-Process & Other Non-Indexed Citations, Ovid MEDLINE® Daily and Ovid MEDLINE®) 1946 to present

Search Strategy:

----

1 exp clinical pathway/ (7266)

2 exp clinical protocol/ (178784)

3 exp consensus/ (16567)

4 exp consensus development conference/ (12448)

5 exp consensus development conferences as topic/ (2968)

6 critical pathways/ (7266)

7 exp guideline/ (36281)

8 guidelines as topic/ (41609)

9 exp practice guideline/ (29146)

10 practice guidelines as topic/ (125684)

11 (guideline or practice guideline or consensus development conference or consensus development conference, NIH).pt. (46006)

12 (standards or guideline or guidelines).ti,kf,kw. (121468)

13 ((practice or treatment* or clinical) adj guideline*).ab. (45501)

14 (position statement* or policy statement* or practice parameter* or best practice*).ti,ab,kf,kw. (38873)

15 (CPG or CPGs).ti. (6054)

16 consensus*.ti,kf,kw. (29948)

17 ((critical or clinical or practice) adj2 (path or paths or pathway or pathways or protocol*)).ti,ab,kf,kw. (23047)

18 recommendat*.ti,kf,kw. (46828)

19 (care adj2 (standard or path or paths or pathway or pathways or map or maps or plan or plans)).ti,ab,kf,kw. (69406)

20 (algorithm* adj2 (screening or examination or test or tested or testing or assessment* or diagnosis or diagnoses or diagnosed or diagnosing or pharmacotherap* or therap* or treatment* or intervention*)).ti,ab,kf,kw. (19893)

21 1 or 2 or 3 or 4 or 5 or 6 or 7 or 8 or 9 or 10 or 11 or 12 or 13 or 14 or 15 or 16 or 17 or 18 or 19 or 20 (659924)

22 exp Henipavirus/ (809)

23 Henipavirus Infections/ (567)

24 (nipah or hendra).tw. (1353)

25 Monkeypox virus/ or Monkeypox/ (496)

26 (monkeypox or "monkey pox").tw. (803)

27 Chikungunya virus/ or Chikungunya Fever/ (3815)

28 Chikungunya.tw. (5982)

29 "Severe Fever with Thrombocyto* Syndrome".tw. (700)

30 SFTS.tw. (1052)

31 Plague/ (5399)

32 plague.tw. (9601)

33 "black death".tw. (284)

34 "pathogen x".tw. (51)

35 22 or 23 or 24 or 25 or 26 or 27 or 28 or 29 or 30 or 31 or 32 or 33 or 34 (20833)

36 21 and 35 (186)

37 limit 36 to yr="2020 - 2021" (30)

Database: Embase 1974 to present

Search Strategy:

----

1 exp clinical pathway/ (8958)

2 exp clinical protocol/ (107994)

3 exp consensus/ (81891)

4 exp consensus development conference/ (24950)

5 exp consensus development conferences as topic/ (24950)

6 critical pathways/ (8958)

7 exp practice guideline/ (615078)

8 guidelines as topic/ (433775)

9 exp practice guideline/ (615078)

10 practice guidelines as topic/ (368202)

11 (guideline or practice guideline or consensus development conference or consensus development conference, NIH).pt. (0)

12 (standards or guideline or guidelines).ti,kw. (149823)

13 ((practice or treatment* or clinical) adj guideline*).ab. (69128)

14 (position statement* or policy statement* or practice parameter* or best practice*).ti,ab,kw. (55950)

15 (CPG or CPGs).ti. (7254)

16 consensus*.ti,kw. (36376)

17 ((critical or clinical or practice) adj2 (path or paths or pathway or pathways or protocol*)).ti,ab,kw. (34345)

18 recommendat*.ti,kw. (56910)

19 (care adj2 (standard or path or paths or pathway or pathways or map or maps or plan or plans)).ti,ab,kw. (121600)

20 (algorithm* adj2 (screening or examination or test or tested or testing or assessment* or diagnosis or diagnoses or diagnosed or diagnosing or pharmacotherap* or therap* or treatment* or intervention*)).ti,ab,kw. (28786)

21 1 or 2 or 3 or 4 or 5 or 6 or 7 or 8 or 9 or 10 or 11 or 12 or 13 or 14 or 15 or 16 or 17 or 18 or 19 or 20 (1018905)

22 exp henipavirus/ (1485)

23 exp Henipavirus infection/ (608)

24 (nipah or hendra).tw. (1533)

25 monkeypox/ or monkeypox virus/ (861)

26 (monkeypox or "monkey pox").tw. (896)

27 Chikungunya virus/ or chikungunya/ (6073)

28 Chikungunya.tw. (7360)

29 "Severe Fever with Thrombocyto* Syndrome".tw. (758)

30 SFTS.tw. (1258)

31 plague/ (6332)

32 plague.tw. (8453)

33 "black death".tw. (287)

34 "pathogen x".tw. (43)

35 22 or 23 or 24 or 25 or 26 or 27 or 28 or 29 or 30 or 31 or 32 or 33 or 34 (24000)

36 21 and 35 (453)

37 36 (453)

38 limit 37 to yr="2020 - 2021" (83)

Database: Global Health <1973 to 2021 Week 39>

Search Strategy:

----

1 exp consensus/ (140)

2 guidelines/ (55586)

3 (standards or guideline or guidelines).ti. (13730)

4 ((practice or treatment* or clinical) adj guideline*).ab. (6597)

5 (position statement* or policy statement* or practice parameter* or best practice*).ti,ab. (5865)

6 (CPG or CPGs).ti. (532)

7 consensus*.ti. (2284)

8 ((critical or clinical or practice) adj2 (path or paths or pathway or pathways or protocol*)).ti,ab. (1890)

9 recommendat*.ti. (7489)

10 (care adj2 (standard or path or paths or pathway or pathways or map or maps or plan or plans)).ti,ab. (6793)

11 (algorithm* adj2 (screening or examination or test or tested or testing or assessment* or diagnosis or diagnoses or diagnosed or diagnosing or pharmacotherap* or therap* or treatment* or intervention*)).ti,ab. (2152)

12 1 or 2 or 3 or 4 or 5 or 6 or 7 or 8 or 9 or 10 or 11 (82545)

13 exp henipavirus/ (1143)

14 (nipah or hendra).tw. (1155)

15 exp monkeypox virus/ (388)

16 (monkeypox or "monkey pox").tw. (489)

17 exp chikungunya virus/ (4437)

18 Chikungunya.tw. (5218)

19 "Severe Fever with Thrombocyto* Syndrome".tw. (564)

20 SFTS.tw. (524)

21 plague/ (3334)

22 plague.tw. (7648)

23 "black death".tw. (109)

24 "pathogen x".tw. (4)

25 13 or 14 or 15 or 16 or 17 or 18 or 19 or 20 or 21 or 22 or 23 or 24 (15014)

26 12 and 25 (185)

27 26 (185)

28 limit 27 to yr="2020 - 2021" (26)

Scopus

((TITLE-ABS-KEY ("clinical pathway*" OR "clinical protocol*" OR consensus OR guideline* OR "position statement*" OR "policy statement*" OR "practice parameter*" OR "best practice*") OR TITLE-ABS-KEY (care W/2 (standard OR path OR paths OR pathway OR pathways OR map OR maps OR plan OR plans)) OR TITLE-ABS-KEY (algorithm* W/2 (screening OR examination OR test OR tested OR testing OR assessment* OR diagnosis OR diagnoses OR diagnosed OR diagnosing)) OR TITLE-ABS-KEY (algorithm* W/2 (pharmacotherap* OR therap* OR treatment* OR intervention*)) OR TITLE (standards OR recommendat*))) AND (TITLE-ABS-KEY (henipavirus OR nipah OR hendra OR monkeypox OR "monkey pox" OR chikungunya OR "Severe Fever with Thrombocyto* Syndrome" OR sfts OR plague OR "black death" OR "pathogen x")) AND (LIMIT-TO (PUBYEAR , 2021) OR LIMIT-TO (PUBYEAR , 2020))

Web of Science Core Collection

#1 TOPIC: ("clinical pathway*" OR "clinical protocol*" OR consensus OR guideline* OR "position statement*" OR "policy statement*" OR "practice parameter*" OR "best practice*" OR CPG OR CPGs) OR TOPIC: ((care near/2 (standard or path or paths or pathway or pathways or map or maps or plan or plans))) OR TOPIC: ((algorithm* near/2 (screening or examination or test or tested or testing or assessment* or diagnosis or diagnoses or diagnosed or diagnosing))) OR TOPIC: ((algorithm* near/2 (pharmacotherap* or therap* or treatment* or intervention*))) OR TITLE: (standards OR recommendat*)

#2 TOPIC: (Henipavirus OR nipah or hendra or monkeypox or "monkey pox" OR Chikungunya OR "Severe Fever with Thrombocyto* Syndrome" OR SFTS OR plague OR "black death" OR "pathogen x")

#3 #2 AND #1

#4 **#1 and #2** and **2020** or **2021** (Publication Years)

The WHO Global Index Medicus Regional Libraries https://pesquisa.bvsalud.org/gim/?lang=en

(tw:("clinical path*" OR "clinical protocol*" or "critical path*" OR "critical protocol*" OR "practice path*" OR "practice protocol*" OR consensus OR guideline* OR standards OR "position statement*" OR "policy statement*" OR "practice parameter*" OR "best practice*" OR CPG OR CPGs OR "care standard" OR "care path*" OR "care map*" OR "care plan*" OR algorithm*)) AND (tw:(Henipavirus OR nipah or hendra or monkeypox or "monkey pox" OR Chikungunya OR "Severe Fever with Thrombocyto* Syndrome" OR SFTS OR plague OR "black death" OR "pathogen x"))

Limits: 2020-2021

Database search results

	**Search results (February 2020)**	**Updated Search results 14/10/2021 (2020-2021 only)**
**Database**	**Cluster 3 results**	
Ovid Medline	157	30
Ovid Embase	382	83
Ovid Global Health	157	26
Scopus	725	131
Web of Science Core Collection	722	176
WHO Global Index Medicus	85	12
TOTAL	2228	458
Total after deduplication	1428	278

Google Scholar – Screened the first 10 pages of results

Google Scholar search updated 18th May 2022

Sorted by relevance:

(guideline|consensus|standards|"clinical path*”|"clinical protocol*"|"practice path*"|" policy statement*"|"best practice*") (monkeypox)

https://scholar.google.co.uk/scholar?q=(guideline%7Cconsensus%7Cstandards%7C%22clinical+path*%E2%80%9D%7C%22clinical+protocol*%22%7C%22practice+path*%22%7C%22+policy+statement*%22%7C%22best+practice*%22)(monkeypox)&hl=en&as_sdt=0,5

S1.2: Grey literature search strategy

The search terms below were used to search for monkeypox clinical management guidelines on the ministry of health, public health, and other relevant websites. The first 50 results were examined. If a relevant guideline was found using the first search term, the other search terms were not used.

Search Terms
Clinical management guidelines for Monkeypox
Monkeypox clinical management guidelines
Monkeypox clinical guidance
Monkeypox guidelines
Monkeypox guidance
Strategies for management of Monkeypox
Clinical practice guidelines for management of Monkeypox
Clinical management of Monkeypox
Monkeypox clinical management

S1.2.1: Websites of organisations searched:

Organisation
Infectious Disease Society of America (IDSA)
European Respiratory Society (ERS)
European society of clinical microbiology and infectious diseases (ESCMID)
British Thoracic Society (BTS)
The National Institute for Health and Care Excellence (NICE)
World Health Organisation (WHO)
US Centre for Disease Control (CDC)
Africa Centre for Disease Control (CDC)
Nigeria Centre for Disease Control (CDC)
China Centre for Disease Control (CDC)
European Centre for Disease Control (CDC)
Medscape
Uptodate guidelines
BMJ Best Practice
Taiwan Centre for Disease Control (CDC)
World Health Organisation regional offices

S1.2.2: Ministry of Health/Public Health countries' websites searched:

Ministry of health countries					
**Latin America**	**Asia**	**Europe**	**Africa**	**Middle East**	**North Americas**
Argentina	Australia	France	Ghana	Saudia Arabia	Canada
Brazil	Japan	Italy	Nigeria	UAE	
Mexico	South Korea	Germany	Cameroon	Egypt	
Jamaica	China Taiwan	Russia	South Africa	Jordan	
Cuba	China	Turkey		Lebanon	
and other countries in the region	Malaysia	Spain		Tunisia	
	Thailand				
	Iran				
	India				
	Indonesia				
	Nepal				
	Bangladesh				
	Pakistan				
	Nepal				

S2: DATA EXTRACTION

S2.1 Data extraction form

Assessment group	Questions
**Availability**	Guideline Title
	Authors
	Issuing Organisation
	Date of latest revision
	Is the guideline adapted from another source?
	Publication type (e.g., website, journal article, etc)
	Intended audience?
	Geographical aim (e.g., China, UK, USA)
	Intended audience
	What is the setting/income classification? (e.g., Low resource setting)
**Inclusivity**	What group of patients is the guideline aimed at?
	Special populations included?
	Is there specific guidance for pregnant patients?
	Is there specific guidance for people living with HIV?
	Is there specific guidance for children?
	Is there specific guidance for older people?
	Are all the above recommendations tied to clear evidence?
	Any other details on population?
**Methodology**	How was the guideline developed?

	Which key stakeholder groups involved?
	How evidence based was each section?
	Are there plans for the guideline to be reviewed?
**Scope**	Any PPE advice given?
	Does the guideline recommend a specific therapeutic intervention (treatment or prophylaxis)?
	Did the guideline provide supportive care recommendations?
	Are routine investigations advised?
	Is there clear advice on symptom management? e.g., analgesia including dose and duration
	Any information regarding vaccination?
	Are empirical antibiotics recommended?
	Any specific therapeutic contraindications?
	Advise tailored to disease severity?
	Notes/other recommendations
	Are all the above recommendations tied to clear evidence?
	Other comments

S3: FIGURES and TABLES

Table S3.1: Quality of the included clinical management guidelines

Guideline	Country/Region	Year	Domain 1 (%)	Domain 2 (%)	Domain 3 (%)	Domain 4 (%)	Domain 5 (%)	Domain 6 (%)	Overall score
**China (MoH)**	China	2003	8	8	6	22	17	0	1
**Dermatology Advisor**	Global	2017	19	19	16	56	15	33	1
**Dermnet**	Global	2014	58	17	16	61	0	8	2
**ECDC**	Europe	2019	6	33	20	53	19	8	1
**Emedicine**	Global	2020	42	31	6	47	10	0	1
**Ireland HPSC**	Ireland	2021	19	22	11	50	21	0	1
**Medscape**	Global	2019	17	44	32	64	8	46	2
**NCDC**	Nigeria	2019	86	3	8	86	13	13	3
**PHE(UKHSA)**	England	2019	28	19	7	44	13	0	1
**Singapore FETP**	Singapore	2020	67	47	20	61	29	0	3
**Taiwan CDC**	Taiwan	2009	19	50	15	44	10	0	1
**UpToDate**	Global	2021	42	47	84	67	8	67	6
**US CDC**	USA	2018	19	22	17	64	21	0	2
**WHO**	Global	2019	6	6	10	50	25	8	1
